# Different Origins of Gamma Rhythm and High-Gamma Activity in Macaque Visual Cortex

**DOI:** 10.1371/journal.pbio.1000610

**Published:** 2011-04-12

**Authors:** Supratim Ray, John H. R. Maunsell

**Affiliations:** Department of Neurobiology & Howard Hughes Medical Institute, Harvard Medical School, Boston, Massachusetts, United States of America; National Institutes of Mental Health, United States of America

## Abstract

During cognitive tasks electrical activity in the brain shows changes in power in specific frequency ranges, such as the alpha (8–12 Hz) or gamma (30–80 Hz) bands, as well as in a broad range above ∼80 Hz, called the high-gamma band. The role or significance of this broadband high-gamma activity is unclear. One hypothesis states that high-gamma oscillations serve just like gamma oscillations, operating at a higher frequency and consequently at a faster timescale. Another hypothesis states that high-gamma power is related to spiking activity. Because gamma power and spiking activity tend to co-vary during most stimulus manipulations (such as contrast modulations) or cognitive tasks (such as attentional modulation), it is difficult to dissociate these two hypotheses. We studied the relationship between high-gamma power, gamma rhythm, and spiking activity in the primary visual cortex (V1) of awake monkeys while varying the stimulus size, which increased the gamma power but decreased the firing rate, permitting a dissociation. We found that gamma power became anti-correlated with the high-gamma power, suggesting that the two phenomena are distinct and have different origins. On the other hand, high-gamma power remained tightly correlated with spiking activity under a wide range of stimulus manipulations. We studied this relationship using a signal processing technique called Matching Pursuit and found that action potentials are associated with sharp transients in the LFP with broadband power, which is visible at frequencies as low as ∼50 Hz. These results distinguish broadband high-gamma activity from gamma rhythms as an easily obtained and reliable electrophysiological index of neuronal firing near the microelectrode. Further, they highlight the importance of making a careful dissociation between gamma rhythms and spike-related transients that could be incorrectly decomposed as rhythms using traditional signal processing methods.

## Abstract 2

High-gamma (80–200 Hz) activity can be dissociated from gamma rhythms in the monkey cortex, and appears largely to reflect spiking activity in the vicinity of the electrode.

## Author Summary

Electrical activity in the brain often shows oscillations at distinct frequencies, such as the alpha (8–12 Hz) or gamma (30–80 Hz) bands, which have been linked with distinct cognitive states. In addition, changes in power are seen in a broad range above ∼80 Hz, called the “high-gamma” band. High-gamma power could arise either from sustained oscillations (similar to gamma rhythms but operating at higher frequencies) or from brief bursts of power associated with spikes generated near the electrode (“spike bleed-through”). It is difficult to dissociate these two hypotheses because gamma oscillations and spiking are correlated during most stimulus or cognitive manipulations. Further, most signal processing techniques decompose any signal into a set of oscillatory functions, making it difficult to represent any transient power fluctuations that occur at the time of spikes. We address the first issue by using a stimulus manipulation for which gamma oscillations and spiking activity are anti-correlated, permitting dissociation. To address the second issue, we use a signal processing technique called Matching Pursuit, which is well suited to capture transient activity. We show that gamma and high-gamma power become anti-correlated, suggesting different biophysical origins. Spikes and high-gamma power, however, remain tightly correlated. Broadband high-gamma activity could therefore be an easily obtained and reliable electrophysiological index of neuronal firing in the vicinity of an electrode.

## Introduction

Neuronal oscillations exist in the brain over a wide range of frequencies, including the delta (1–3 Hz), theta (4–8 Hz), alpha (9–12 Hz), beta (12–30 Hz), and gamma (30–80 Hz) bands, and are thought to reflect cortical processing [1]. In addition to the modulation of power in some of the aforementioned frequency bands, there is often an increase in power in a broad frequency range above 80 Hz, called the “high-gamma” band (80–200 Hz). This increase in high-gamma power has been most consistently observed in electrocorticogram (ECoG) studies in humans [2]–[5] but is also observed in local field potentials (LFPs; [6]–[8]) and magnetoencephalogram (MEG; [9],[10]). It has been seen in several cortical areas, under diverse stimulus conditions and a range of cognitive states (for a review see [11]).

The functional significance of high-gamma activity and its relationship to gamma rhythms that are typically observed at lower frequencies (30–80 Hz) remain unclear. One suggested role of the gamma band is to provide communication channels between cortical areas [12],[13]. Within this framework, there could be multiple frequency bands for communication [14], so the high-gamma band could serve as a distinct channel [15], possibly nested within a low frequency rhythm [3],[5],[16]. On the other hand, several studies have shown that spiking activity is coupled to power in the high-gamma range [6]–[8],[17]–[19]. Because under many conditions the gamma power and firing rates are correlated (for example, during attentional modulation), it is difficult to distinguish between the two possibilities described above.

We addressed this issue by studying the LFP power spectrum in V1 of monkeys while manipulating the stimulus size, because increasing the size decreases the firing rate but increases the strength of the gamma rhythm (i.e., the two are anti-correlated; [20]), permitting a dissociation. Using a signal processing technique called Matching Pursuit (MP) that imposes minimal a priori assumptions on LFP decomposition and can simultaneously resolve both transient and oscillatory components in the LFP [8],[18], we studied the relationship between spiking activity and high-gamma power under conditions when the gamma rhythm was absent (no stimulus), weak (small stimulus size), or strong (large size). We found that high-gamma activity was strongly correlated with the multiunit spiking, under different manipulations of stimulus size and temporal frequency. Our results show that multiunit activity can be reliably estimated from the high-gamma power. Further, while investigating the role of high-gamma band in communication or coding, it is important to account for population spiking activity because it may also modulate power in the high-gamma band.

## Results

Recordings were made from an array of 96 electrodes (Blackrock Systems) that was chronically implanted in the right hemisphere of V1 in two monkeys. The receptive fields were in the lower left visual quadrant at an eccentricity of 3–5°. The monkeys performed an orientation change detection task (Figure S1A), where they attended to a Gabor stimulus *outside* the receptive field while a series of gratings of six different sizes and orientations were presented inside the receptive field of one of the recording sites (new location for each session) for 400 ms with an interstimulus interval of 600 ms (see Materials and Methods for further details). Analysis was restricted to sites whose receptive field centers were within 0.2° from the stimulus center and for which the firing rate was at least 1 spike/s for each of the six sizes. This yielded 15 and 104 sites from Monkeys 1 and 2. Unless stated otherwise, the results shown below were obtained after pooling the data across orientations to increase the statistical power, although similar results were obtained when the analysis was performed only on the preferred orientation.

Four analyses were performed. First, we studied the correlation between firing rates and LFP power (as a function of frequency) while varying the stimulus size. For this analysis, firing rates and LFP power were averaged over time (between 200 and 400 ms after stimulus onset) as well as over stimulus repetitions. Second, we computed the correlation between two time-series: firing rates and the average LFP power in different frequency bands, both computed in 2 ms bins and averaged over stimulus repetitions. Third, we computed the trial-by-trial co-variability in firing rates and LFP power in different frequency bands under identical stimulus conditions. Finally, we performed a spike-triggered analysis in two dimensions (time and frequency) to estimate the temporal and spectral components in the LFP that were locked to spikes. Spectral analyses were performed using the MP algorithm (see Materials and Methods for details) and were compared with the more traditional multitaper method [21],[22] in the Supporting Information section.

### Correlation between Firing Rates and LFP Power as a Function of Frequency

**Figure 1:**
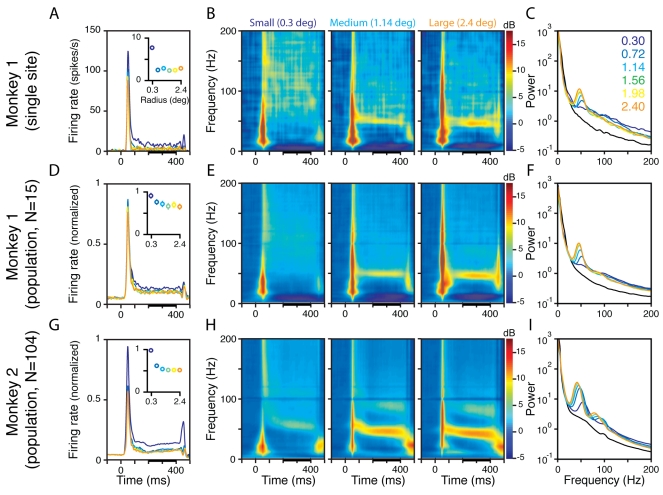
Dissociation of the gamma rhythm and high-gamma activity by manipulating stimulus size. (A) Average multiunit recorded from a single site in Monkey 1 during the presentation of a static grating (0 to 400 ms) at six different sizes, shown in different colors. The inset shows the average firing rate between 200 and 400 ms, indicated by a thick black line on the abscissa. (B) Time-frequency energy difference plots (in dB) showing the difference in energy from baseline energy (−300 to 0 ms, 0 denotes the stimulus onset, difference computed separately for each frequency) for the smallest (radius of 0.3°, left panel), medium (1.14°, middle), and largest (2.4°, right) sizes. The gamma rhythm at ∼50 Hz increases with size, while the high-gamma activity above the gamma band decreases with size. (C) The LFP energy between 200 and 400 ms (denoted by a thick black line on the abscissa in B) as a function of frequency for the six sizes, whose radii are listed in the legend. The black line shows the LFP energy in the baseline period. (D–F) and (G–I) show corresponding population responses of 15 and 104 sites from Monkeys 1 and 2, respectively. For (D) and (G), the responses are normalized by dividing by the maximum firing rate for each site. Monkey 2 showed two distinct gamma bands at ∼50 and ∼90 Hz.

Figure 1A shows the average multiunit firing rate of a typical recording site from Monkey 1 when gratings of six different radii (values shown in the inset of Figure 1C) were presented between 0 and 400 ms. The inset shows the firing rate between 200 and 400 ms (thick horizontal black line on the time axis), as a function of stimulus size. As expected, increasing the stimulus size increased the strength of the inhibitory surround, which decreased the firing rate. Figure 1B shows the change in LFP power relative to a baseline period (defined as 0 to 300 ms before stimulus onset) for three different sizes (radii of 0.3°, 1.14°, and 2.4°, shown in Figure S1B). These time-frequency energy difference spectra showed a large broadband increase in power in the first 100 ms after stimulus onset, coinciding with the transient increase in firing rate (Figure 1A). The gamma rhythm, represented by a horizontal band at ∼50 Hz in the time-frequency spectrum, appeared after the initial transient and continued until the stimulus was turned off at 400 ms. Consistent with the results shown in [20], gamma rhythm amplitude increased with increasing stimulus size. We also observed an increase in power over a broad frequency range above the gamma range (>60 Hz). However, power in this band showed the opposite trend—it decreased with increasing stimulus size, similar to the decrease observed in the firing rates. Figure 1C shows the energy between 200 and 400 ms (indicated by thick black lines on the time axes of Figure 1B), as a function of frequency, for the six stimulus sizes (colored traces) as well as the pre-stimulus baseline (black trace). While the power in the gamma range (40–60 Hz, peak at ∼50 Hz) increased with size, beyond the gamma range there was a clear decrease in power with increasing size. Figure 1D–F and G–I show the population average of LFP recordings from all the sites in Monkeys 1 and 2 (15 and 104, respectively). The firing rates were normalized by dividing by the maximum firing rate for each site before averaging (Figure 1D and G). The time-frequency power difference spectra (Figure 1E, H) and the power versus frequency spectra (Figure 1F, I) were averaged across sites on a log scale (see Materials and Methods for details). Note that Monkey 2 showed a second gamma rhythm at ∼90 Hz (also observed by [20]), and therefore the relative decrease in LFP power with increasing stimulus size could be observed only above ∼100 Hz.

Although gamma rhythm and high-gamma activity are usually distinguished solely on the basis of frequency (30–80 Hz versus 80–200 Hz), it is critical to note that these two phenomena have very different spectral profiles and there could be considerable overlap between the frequency ranges. The gamma rhythm is “band-limited,” with a bandwidth of ∼20 Hz, and is visible in the power spectrum as a distinct “bump.” Typically the center frequency of gamma rhythm is between 30 and 80 Hz, but occasionally there is a second peak at higher frequencies also (Monkey 2). In contrast, high-gamma activity is “broadband,” represented by an elevation in power over a broad frequency range without any obvious bumps. Although high-gamma activity is more prominent at frequencies above ∼80 Hz, it can be observed at frequencies as low as ∼50 Hz when gamma rhythm is absent (see the “*spike-triggered average analysis*” section below).

**Figure 2 pbio-1000610-g002:**
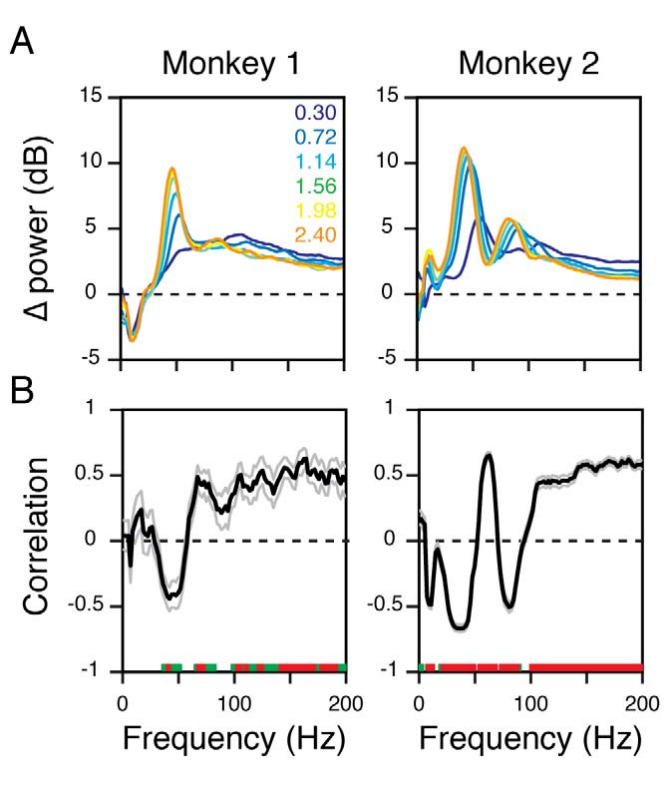
Correlations between power and firing rates have different signs in gamma versus high-gamma bands. (A) Average relative change in power between 200 and 400 ms from baseline power (difference between the colored traces and the black trace in Figure 1F and 1I), for 15 and 104 sites in Monkeys 1 (left panel) and 2 (right panel). Radii are listed again in the legend for clarity. (B) Spearman rank correlation between the six power values (one for each size) at each frequency and the six firing rates values, computed individually for each site and then averaged. Black and gray traces show the mean and SEM of 15 and 104 sites in the two monkeys. The correlation values significantly different from zero are shown in green (*p*<0.01, uncorrected) and red (*p*<0.05 with Bonferroni correction).

Figure 2A shows the mean change in power (200–400 ms after stimulus onset) from baseline, as a function of frequency (obtained by subtracting the black trace from the colored traces in Figure 1F and 1I). To relate the changes in power with stimulus size with corresponding changes in firing rates, we computed the Spearman rank correlation, for each site and at each frequency, between the six power values and firing rate values (one value for each stimulus size, all values computed between 200 and 400 ms after stimulus onset). Figure 2B shows the mean (solid black line) and SEM (gray line) of the Spearman rank correlation of 15 and 104 sites in Monkeys 1 and 2, as a function of frequency. Correlation values significantly different from zero are shown in green (*p*<0.01 without Bonferroni correction, *t* test) and red (*p*<0.05 with Bonferroni correction, *t* test). For Monkey 1, the correlation was significantly negative in the gamma range but became positive above ∼60 Hz. For Monkey 2, a negative correlation between power and firing rates was observed at both the gamma bands (30–50 Hz and 80–95 Hz). Further, due to a shift in the peak gamma frequency with stimulus size [20], power between 50 and 80 Hz showed a positive correlation. For both monkeys, the correlation between firing rates and LFP power beyond 100 Hz was consistently positive.

LFP energy was averaged between 200 and 400 ms to avoid stimulus-induced transients, which were prominent in the first 100 ms after stimulus onset (Figure 1B,E,H). Under these circumstances, multitaper method is expected to yield similar results, which was indeed the case (Figure S2).

**Figure 3:**
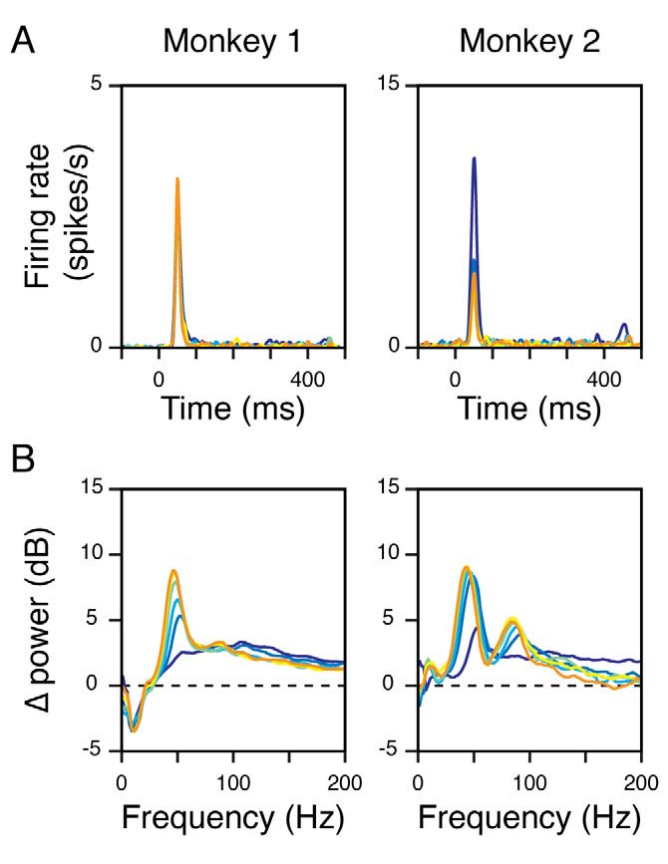
Changes in power with stimulus size are observed even when firing rates are negligible. (A) Average firing rate of 30 and 10 sites in Monkeys 1 (left column) and 2 (right column), for which less than 0.5 spikes/s were obtained between 200 and 400 ms. (B) Difference in power between 200 and 400 ms from baseline power (same format as Figure 2A) for these sites.

The positive correlation between spiking activity and LFP power above 100 Hz could be due to “spike bleed-through,”—that is, energy associated with action potentials of the neurons near the microelectrode bleeding into the low frequency range. One possibility is that only the neurons very close to the microelectrode, whose action potentials are large enough to be isolated using an amplitude threshold, contribute to the high-gamma power. However, we observed an increase in LFP power above 100 Hz even in sites where the firing rates of isolated neurons were negligible or even decreased after stimulus onset. Figure 3A shows the firing rates of 30 and 10 sites in Monkeys 1 and 2, for which the firing rate between 200 and 400 ms was less than 0.5 spikes/s. Figure 3B shows the average change in power between 200 and 400 ms from baseline for these sites. These plots show the same trend as Figure 2A, even though there were almost no isolated spikes recorded during this period. This suggests that high-gamma power reflects the firing of a larger pool of neurons near the microelectrode than those that are resolved from the background.

### Correlation between the Time-Series of Firing Rates and LFP Power

**Figure 4:**
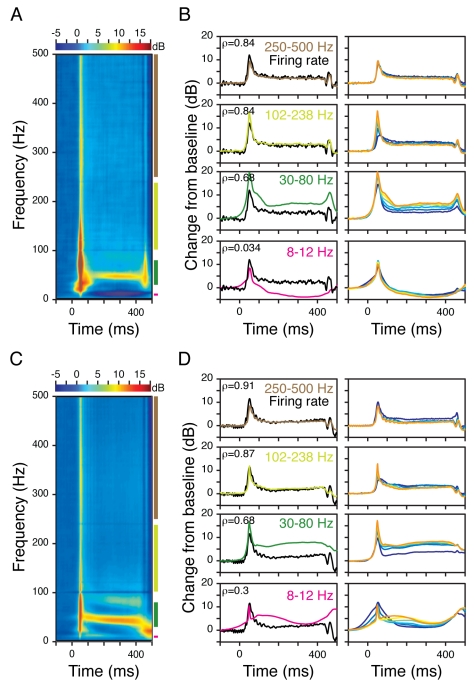
Correlations between the time-courses of firing rate and LFP power in different frequency bands. (A) The mean time-frequency energy difference plot (in dB) of 15 sites from Monkey 1, when the largest stimulus is presented. Same as the right panel of Figure 1E, except that the displayed frequency range is up to 500 Hz. The vertical colored lines in the right mark the four frequency bands used for analysis—alpha (8–12 Hz; magenta), gamma (30–80 Hz; dark green), high-gamma (102–238 Hz, excluding 118–122 Hz; light green), and 250–500 Hz (brown). (B) Panels in the left column show the relative change in LFP power in the four frequency bands (colored traces) for the largest stimulus, along with the relative change in firing rate (black trace, same for all panels). The Spearman rank correlation between the two traces is denoted in the upper-left corner. Panels in the right show the relative change in LFP power for different stimulus size (same color code as Figures 1 and 2, the orange trace is the same as the colored trace in the left column). (C, D) Same as (A, B) for 104 sites in Monkey 2.

The analysis described above shows the correlation in firing rates and LFP power over a 200 ms interval. However, if the LFP power above 100 Hz indeed reflects the spiking activity of a population of neurons, it should be correlated with the multiunit firing rate at a finer timescale, such that the two time-series should covary. Figure 4A and 4C show the mean change in power spectrum for all the sites in Monkeys 1 and 2 for the largest stimulus (same as the right column of Figure 1E and 1H, but the displayed frequency range is up to 500 Hz). We divided the LFP power into four bands—8–12 Hz (alpha band), 30–80 Hz (gamma band), 102–238 Hz (high-gamma band; the lower cutoff is above 100 Hz to avoid the second gamma peak in Monkey 2), and 250–500 Hz—and computed the power in these bands as a function of time (the bands are shown in different colors in the right side of the time frequency plots). We observed three small noise peaks in our LFP data at 100 Hz (monitor refresh rate) and the second and fourth harmonic of line noise (120 and 240 Hz), so for the computation of the high-gamma power we excluded the power between 118 and 122 Hz.

The left columns of Figure 4B and 4D show the mean change in power from baseline in the four frequency bands described above (colored traces), along with the mean change in firing rate from baseline (black trace, same for all panels), for all sites. The Spearman rank correlation between the two curves is shown at the top left corner. While changes in power in the alpha or gamma band were not well correlated with the changes in firing rate, we observed a strong correlation in the dynamics of high-gamma power and firing rate. We also observed a strong correlation between firing rates and LFP power between 250 and 500 Hz, which is expected because of spike bleed-through in this frequency range. Similar results were obtained for other stimulus sizes, or when gamma range was taken between 40 and 70 or 30 and 60 Hz (unpublished data). The right columns of Figure 4B and 4D show the changes in power in the four bands for the six stimulus sizes. As expected, we observed dissociation in gamma versus high-gamma power—while the power in the gamma band increased more for a large stimulus than a small one, power in the high-gamma band showed the opposite trend.

Similar trends were observed when the Spearman correlation was computed between firing rate and power curves obtained from individual sites, although the correlation values were smaller. For power between 250 and 500 Hz, the median ± SE (estimated using bootstrapping) correlation values for Monkeys 1 and 2 were 0.74±0.01 and 0.72±0.01. For the high-gamma range, the median correlations were 0.67±0.01 and 0.61±0.02, while for the gamma range the median correlations were 0.56±0.01 and 0.47±0.01.

The ability of the MP algorithm to capture the broadband transient after stimulus onset (between 0 and 100 ms in Figure 4A and 4C) is critical for the tight correlation between firing rates and power at higher frequencies. Figure S3 shows similar analysis using the multitaper method. Even with small window size (64 ms), for which the gamma rhythm is not well represented due to poor spectral resolution (Figure S3B and S3D), correlations between firing rates and power at high-gamma frequencies and above were smaller than the correlations obtained with MP.

To account for possible time lags between firing rate and power at different frequency bands, we also computed the Spearman rank correlation after first shifting the firing rate curve by a small duration (see Materials and Methods for details). The correlations varied only slightly as a function of the lag and typically were highest near time zero (no lag).

**Figure 5:**
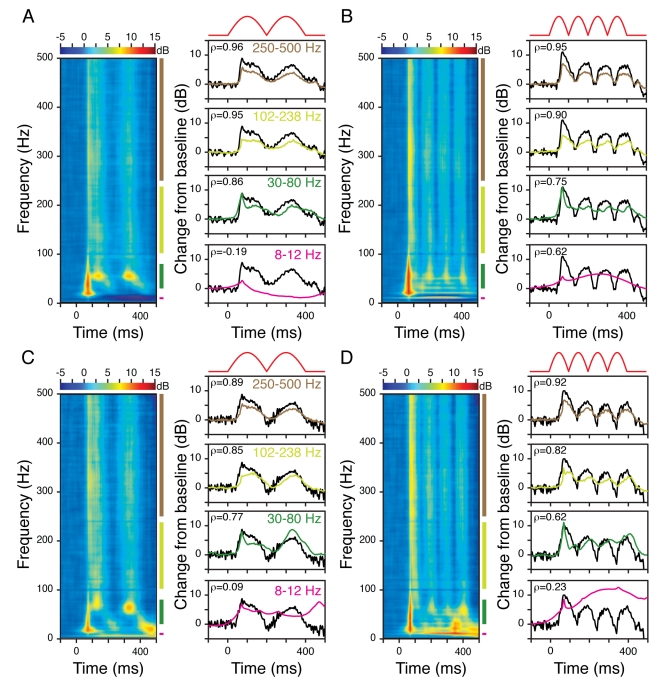
Correlations between firing rate and LFP power in different frequency bands for stimuli with different temporal frequency profiles. (A) The left panel shows the average time-frequency energy difference spectrum of 19 sites in Monkey 1 when the stimulus was presented with a counter-phasing temporal frequency of 2.5 Hz. The contrast profile is shown in red on top of the right panels. The right panels show the relative change in power in different frequency bands as well as in the firing rates, as a function of time. Same format as in Figure 4. The Spearman correlation values between the firing rate and power traces are shown in the top left corner. (B) Same as panel (A), for a temporal frequency of 5 Hz. (C, D) Same as (A, B) for 66 sites in Monkey 2.

Because our stimuli were static gratings, both the firing rates and power in different frequency bands showed a pronounced transient response before reaching a steady state after ∼100 ms. We next asked whether the LFP power above 100 Hz could track the changes in firing rate if the rate changed periodically during stimulus presentation. To test this, we used a different dataset in which the temporal frequency of the stimulus was varied in a sinusoidal counter-phasing fashion (i.e., a static grating with contrast varying in a sinusoidal manner; temporal frequency was varied across stimulus presentations). Figure 5A and 5C show the average LFP power difference (left panel) as well as changes in power in different frequency bands (right panels, colored traces) along with changes in firing rates (right panels, black traces, same for all the panels) of 19 and 66 sites from Monkeys 1 and 2 when the temporal frequency of the stimulus was 2.5 Hz (contrast profile is shown in red above the top right panel; we get two peaks in the contrast profile per cycle). Firing rates followed the contrast profile and showed a periodic modulation at twice the temporal frequency (5 Hz). The center frequency of the gamma rhythm was dependent on the instantaneous contrast [23]. However, the power above the gamma range (>100 Hz) followed the same pattern as the firing rates, with a Spearman correlation of more than 0.85 (shown at the upper-left corner of each plot). Similar trends were observed for a temporal frequency of 5 Hz (Figure 5B and 5D) as well as 10 and 20 Hz for Monkey 2 (Figure S4). Beyond 20 Hz, the firing rates did not follow the temporal frequency and the correlation estimates were noisier. These results agree well with our earlier observation that LFP power above ∼100 Hz closely tracks the changes in firing rates. Note that at temporal frequencies of 5 Hz and above, alpha and gamma bands contained harmonics of the stimulus frequencies, which made their estimation of power inaccurate.

### Trial-by-Trial Co-variations in Firing Rates and LFP Power

**Figure 6:**
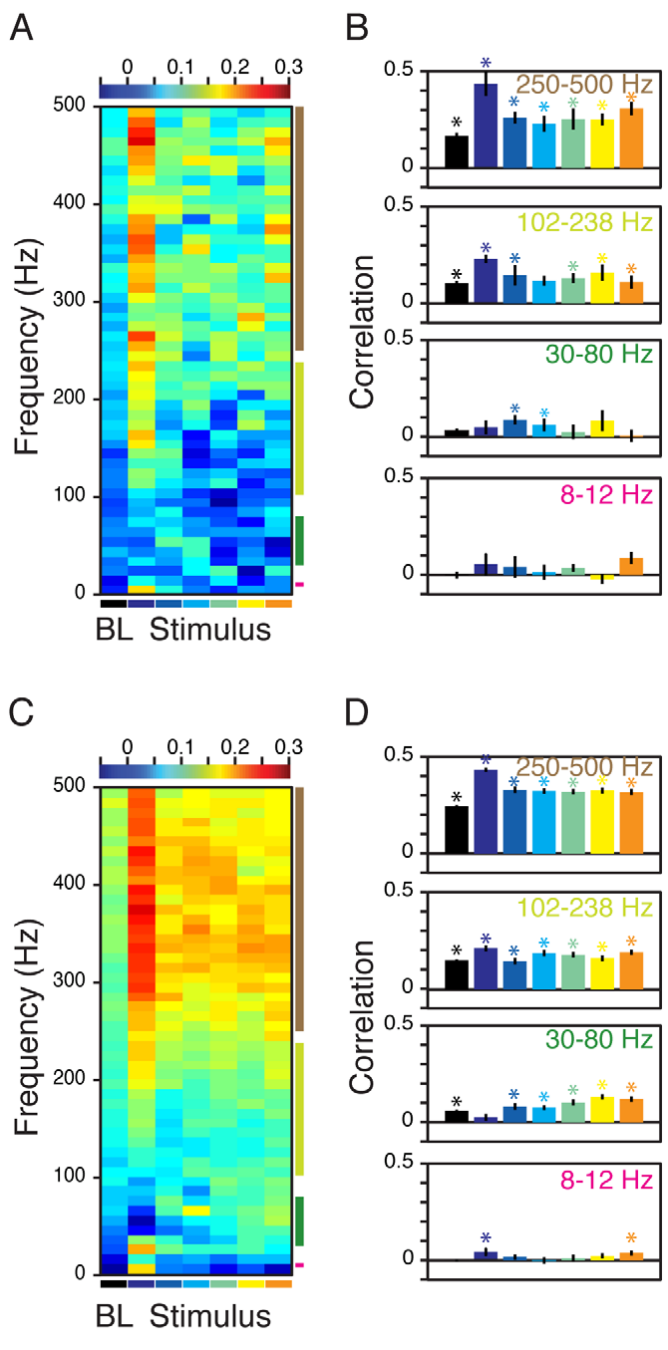
Trial-by-trial Spearman correlation between firing rates and LFP power at different frequencies when stimulus conditions are identical. (A) The median Spearman rank correlation between LFP power at different frequency bins (size of 25 Hz, computed in steps of 10 Hz) and firing rates, both computed between 200 and 400 ms after stimulus onset, for 15 sites in Monkey 1. The correlations were computed separately for each size, site, and orientation, so that the stimulus conditions were identical. The first column shows the median correlations during the pre-stimulus period (denoted “BL” for baseline). The remaining six columns represent the six stimulus sizes (denoted by the respective color below the *x*-axis). (B) Median Spearman correlation, computed for the four frequency bands used in Figures 4 and 5. Correlations significantly different from zero (*p*<0.05, Bonferroni corrected) as shown with asterisks. (C,D) Same as (A, B) for 104 sites in Monkey 2.

In the previous analyses we studied the relationship between LFP power and firing rates under different stimulus conditions (different sizes). Under these circumstances, it is difficult to determine whether the changes in firing rates and LFP power are due to the same biological mechanism, because changing the stimulus may lead to several changes in the neuronal network. A partial way to address this concern is to study the trial-by-trial covariation in firing rates and LFP power in different frequency bands when the stimulus conditions are identical across trials. For this analysis, we first computed the firing rates and LFP power between 200 and 400 ms for each stimulus presentation. LFP power was computed in a 25 Hz band, in steps of 10 Hz. The Spearman rank correlation between firing rate and LFP power at each frequency was computed individually for each site, orientation, and size. Similar analysis was also performed before stimulus onset (−300 to 0 ms). Figure 6A and 6C show the median Spearman rank correlation, averaged across days and orientations, for Monkeys 1 and 2. The first column shows the correlation during the baseline period (indicated by a black horizontal line below the *x*-axis; the correlation values were averaged across sizes). The other columns show the correlation during the stimulus period (each column represents a different size, indicated by a colored line below the *x*-axis). Correlation became stronger with increasing frequency during baseline as well as stimulus period. The smallest stimulus (second column) that produced the highest firing rate had the highest correlation, which is expected because correlations are difficult to detect when firing rates are low.

To compare the correlation in gamma versus high-gamma bands, we averaged the LFP power in the four bands used in Figures 4 and 5 and computed their trial-by-trial correlation with firing rates (Figure 6B and 6D). Correlation values significantly different from zero (*p*<0.05 after Bonferroni correction, rank sum test) are indicated by asterisks. For the alpha band, the correlations were very small and rarely significant (medians averaged over all sizes were 0.03±0.01 and 0.02±0.005 for the two monkeys). For the gamma band, the overall medians were 0.05±0.01 and 0.09±0.006, typically not significant for Monkey 1 but significant for Monkey 2. However, for the high-gamma band, the correlations were significant at all sizes for both monkeys, except for the radius of 1.14 (cyan bar) for Monkey 1, for which the uncorrected *p* value was 0.02. The overall median correlations were 0.14±0.01 and 0.18±0.006 for the two monkeys. The largest correlations were obtained for power between 250 and 500 Hz, with medians of 0.28±0.01 and 0.34±0.007, highly significant for all stimulus sizes. Similar results were obtained during the baseline period (black bars). These results suggest that firing rates are more strongly correlated with LFP power at progressively higher frequencies. Importantly, this correlation can be observed even at frequencies as low as the high-gamma range—that is, ∼100 Hz. These results are consistent with the temporal correlation analysis shown in Figures 4 and 5, which also showed larger correlations between firing rates and LFP power at higher frequencies.

The results were similar when the same analysis was done using the multitaper method (Figure S5), which is expected because the analysis period was either before stimulus onset or after the response transient.

### Spike-Triggered Average Analysis

**Figure 7:**
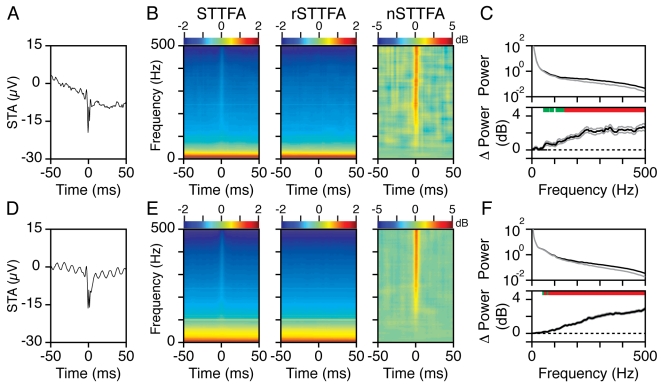
Spike-triggered average in time-frequency domain during baseline period. (A) The mean spike-triggered average from spikes taken between 268 and 132 ms before stimulus onset, from the 14 sites for which at least 25 spikes were obtained. (B) Left panel shows the spike-triggered time-frequency average (STTFA), computed by averaging short 2-D segments of the time-frequency energy spectrum centered on the spikes. The middle panel shows the STTFA computed after first randomizing the spike times (called rSTTFA). The panel in the right shows the relative change in the time-frequency spectrum locked to the spike, computed by taking the difference between log(STTFA) and log(rSTTFA) (called the normalized STTFA, or nSTTFA). (C) The mean energy between −1 to 3 ms of the STTFA (black) and the rSTTFA (gray), as a function of frequency (upper plot). The difference between the two is shown in the lower panel (mean in black, SEM in gray). The values significantly different from zero are shown in green (*p*<0.01, uncorrected) and red (*p*<0.05 with Bonferroni correction). (D–F) Same as (A–C), for 103 sites in Monkey 2.

The previous two analyses show that LFP power becomes more correlated with spiking activity with increasing frequency, and importantly, this correlation is significant even in the high-gamma range. In this section we characterize this correlation in more detail by studying the LFP around the time when an action potential was recorded. A commonly used measure is the spike-triggered average (STA) of the LFP, which is computed by taking small segments of the LFP around each spike followed by averaging. Figure 7A and 7D show the mean STA of 14 and 103 sites from which at least 25 spikes were obtained during the baseline period (268 to 132 ms before stimulus onset) for Monkeys 1 and 2. The STA revealed a sharp negative peak at time zero, which is due to the sodium influx into the neuron. For Monkey 2, the STA also showed an oscillatory component at 100 Hz (refresh rate of the monitor). The STA, however, provides no information about the frequency content of the spike-locked events in the LFP. To study the relationship between spikes and LFP in the time-frequency domain, we computed the spike-triggered time-frequency average (STTFA), where we took small 2-D segments from the time-frequency energy spectrum centered on the spikes and averaged those segments (for details and discussion of this method, see [18]). Left panels of Figure 7B and 7E show the STTFAs during the baseline period for Monkeys 1 and 2. Note that these STTFAs show a dominant 1/f power spectrum. This is because unlike the STA, for which any signal component not phase locked to the spike cancels out with averaging, the STTFA averages segments of the energy spectrum, which are always positive and hence do not cancel out. The STTFA therefore shows both the component locked to the spikes and the usual 1/f power spectrum that is not locked to the spike. This second component can be estimated by randomizing the times at which the STTFA is computed, irrespective of the occurrence of spikes. The randomized STTFA, called “rSTTFA,” is shown in the middle panels of Figure 7B and 7E. The components locked specifically to the spikes are obtained by taking the difference of log(STTFA) and log(rSTTFA), called the normalized STTFA (nSTTFA), shown in the right panels of Figure 7B and 7E. Because we subtract the log of powers, the nSTTFA shows the ratio of powers of STTFA and rSTTFA, at each time-frequency bin, on a log scale.

The nSTTFA is not the time-frequency power spectrum of the STA. The power spectrum of the STA (unpublished data) has power at very low frequencies as well, which is expected because it can be approximated by a Gaussian function with a small sigma, whose Fourier Transform also has a Gaussian profile (with a large sigma). However, at lower frequencies the STA power is masked out by the much larger “1/f” noise present in the LFP. The nSTTFA shows the portion of the power locked to the spike that is larger than the 1/f noise.

We found that most of the energy due to the spiking activity was locked to a narrow time window around the time of the spike, temporally coinciding with the sharp transients shown in Figure 7A and 7D. We also observed that the nSTTFA power was visible down to frequencies as low as ∼50 Hz. To quantify this, we averaged the power in the STTFA and rSTTFA between −1 to 3 ms around the time of the spike and plotted the power as a function of frequency. The upper panels in Figure 7C and 7F show the power of the STTFA (black trace) and rSTTFA (gray trace) between −1 and 3 ms, as a function of frequency. The lower panels show the difference between these two traces (black line), along with the SEM (gray traces). The values that are significantly different from zero are shown in green (*p*<0.01, no Bonferroni correction, *t* test) and red (*p*<0.05 with Bonferroni correction, *t* test). We defined the “cutoff frequency” as the frequency above which 10 consecutive frequency bins were significant at *p* = 0.01 (without correction). This cutoff frequency is crucial because it indicates the frequency limit above which spikes can significantly affect the LFP power. For the two monkeys, the cutoff frequencies were 52 and 48 Hz. Thus, the spike energy can be observed in the LFP power spectrum at frequencies as low as 50 Hz. A visual inspection of the nSTTFA shows that the spike energy was very prominent above ∼100 Hz.

**Figure 8:**
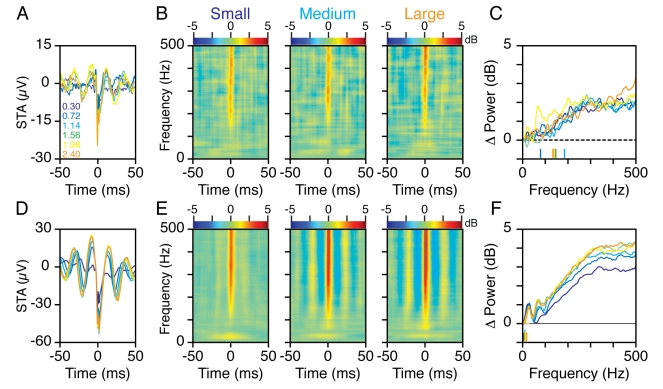
Spike-triggered average in time-frequency domain during stimulus presentations. (A) Mean spike-triggered average of 15 sites for Monkey 1 for which at least 25 spikes were available between 232 and 368 ms after stimulus onset, for the six stimulus sizes. (B) The normalized STTFA (see text and Figure 7 for details) when a small (left), medium (middle), and large (right) stimulus was presented. (C) The difference between the mean energy between −1 and 3 ms of the STTFA and rSTTFA (same as the lower panel of Figure 7C), for the six stimulus sizes. The horizontal lines at the bottom indicate the “cutoff frequency” for each stimulus size (see text for definition). (D–F) Same as (A–C), for 94–103 sites in Monkey 2 for which at least 25 spikes could be obtained. The number of sites decreases from 103 to 94 because the firing rates decrease with increasing stimulus size.

Similar results were obtained when the analysis was done during the stimulus period. Figure 8A and 8D show the mean STA of 15 sites for Monkey 1 and 94–103 sites for Monkey 2, from which at least 25 spikes could be obtained between 232 and 368 ms after stimulus onset, for the six stimulus sizes. The STA revealed that spikes occurred at preferential phases of the gamma rhythm, which was also observed in the spike-field coherence (SFC; Figure S6). The STTFA (unpublished data) also showed a band-limited elevation in power in the gamma range with increasing stimulus size, but this was not observed in the nSTTFA (Figure 8B and 8E). This is expected because the gamma band had elevated power throughout the analysis period, which was also picked up in the rSTTFA and hence was subtracted out in the nSTTFA. Similar to the nSTTFA obtained during the baseline (Figure 7B and 7E, right column), the nSTTFA during the stimulus period showed a prominent burst of power beyond 100 Hz around a small window near time zero. In addition, for Monkey 2 we observed alternating bands of high and low energy in the high-gamma range in the nSTTFA (Figure 8E, middle and right panels), with bands of high energy coinciding with the troughs of the gamma rhythm seen in Figure 8D. This was also expected, because spikes preferentially occurred at the trough of the gamma rhythm (at 0 ms and about ±25 ms) and each high-gamma burst (vertical red/yellow band) reflected this enhancement of spiking activity. Similarly, firing rates were lower than usual during the peaks of the gamma cycle, which were reflected as bands of low energy (vertical blue bands). Also, the degree of phase locking increased with increasing stimulus size for Monkey 2 (Figure S6C), which made the high-gamma power fluctuations more prominent. This effect was not observed in Monkey 1, where the degree of gamma phase locking was much weaker and the SFC did not increase significantly with stimulus size (Figure S6A).

Figure 8C and 8F show the mean difference in the log of power between the STTFA and rSTTFA energy averaged between −1 and 3 ms, as a function of frequency, for Monkeys 1 and 2 (analogous to the plots shown in the lower panels of Figure 7C and 7F). The “cutoff” frequencies as defined before are indicated by short vertical lines near the bottom. For Monkey 1, these traces are much noisier than the baseline condition shown in Figure 7C because for the baseline condition we used spikes before the presentation of all sizes. The cutoff frequencies ranged from 80 to 185 Hz. For Monkey 2, we observed a small but significant increase in energy at very low frequencies, possibly reflecting synaptic events associated with synchronous activity, so the cutoff frequencies were ∼10 Hz (this small peak can also be observed in Figure 8E). Nevertheless, plots 8C and 8F, as well as the nSTTFA plots shown in 8B and 8E, showed a reflection of spiking activity in the LFP that became progressively more prominent between 50 and 300 Hz before reaching a plateau.

MP algorithm is crucial for this analysis, because it readily accommodates sharp transient-like fluctuations like those associated with spikes. If the time-frequency LFP energy is computed using the multitaper method instead, all functions have a fixed spread in time and frequency (depending on the window length). Figure S7 shows similar analysis using multitaper method. Even with very short windows, the resolution is much worse than MP because most of the energy associated with spiking is spread out over the width of the window.

## Discussion

We show that the broadband increase in power most consistently observed above ∼80 Hz in the LFP can be dissociated from the band-limited gamma rhythm, which typically has a center frequency between 30 and 80 Hz and a bandwidth of ∼20 Hz. Further, high-gamma activity is tightly correlated with the firing rates of neurons near the microelectrode. When the gamma rhythm is weak or absent, a substantial correlation can be observed between the spiking activity and LFP power at frequencies as low as ∼50 Hz.

All the results can be explained as follows: spikes are associated with a sharp transient in the LFP signal (Figures 7A, 7D, 8A, 8D), which has power in a broad frequency range (including very low frequencies). However, at lower frequencies it is masked out by a much larger “1/f” noise. Therefore, the energy associated with spiking can only be readily observed when it exceeds the 1/f noise (the “cutoff frequency” described above). We show that this cutoff frequency is ∼50 Hz and the spike energy becomes more prominent with increasing frequency (Figures 7C, 7F, 8C, 8F). This explains the increase in correlation between firing rates and LFP power at high frequencies, when computed on a broad timescale (200 ms) with different stimulus conditions (Figures 1–3) as well as on a trial-by-trial basis under identical stimulus conditions (Figure 6). This also explains a tight co-variation between the firing rate curve and LFP power curve at higher frequencies (Figures 4 and 5). We also show that spikes that are too small to be isolated contribute to the high-gamma power (Figure 3). Thus, high-gamma is a useful measure of the average population firing near the microelectrode.

We emphasize here that the time-frequency components locked to spikes—that is, the high-gamma burst shown in Figures 7 and 8, only describe the extracellular changes in field potential when an action potential is observed and may include not only the changes due to the action potential itself but also other changes in the network that might be related to spiking activity (such as synaptic input that leads to the spike). In other words, we cannot directly associate the time-frequency components shown in Figures 7 and 8 with specific currents that are associated with action potentials. That would require similar analysis on intracellular recordings with specific channel blockers.

### High-Gamma Activity in the Brain

Although the broadband increase in power in the high-gamma range reported in this article has been observed in many studies (for a review see [11]), there is also evidence of characteristic oscillations at high frequencies. For example, in the CA1 hippocampal region, Buzsaki and colleagues reported the existence of very fast network oscillations in the range of ∼200 Hz (also called “ripple” oscillations) during behavioral immobility, consummatory behaviors, slow-wave sleep, and exploratory behavior [24],[25]. Similarly, Barth and Jones described ultra-fast oscillations (>200 Hz) in the rat barrel cortex [26]–[28]. Indeed, the oscillation frequency of a network critically depends on excitation-inhibition balance [23],[29],[30] and could exceed 100 Hz [29]. Therefore, 100 Hz should not be thought of as a “strict boundary” separating oscillatory and broadband activity.

Could the broadband high-gamma power reflect many narrowband oscillators operating at many different center frequencies? While this possibility is difficult to rule out completely, several problems arise with this hypothesis. First, the MP algorithm has different types of functions to represent oscillatory and transient signals; the type of function chosen depends on the properties of the signal itself. We found that most of the energy in the high-gamma range is captured by transient functions, which further are tightly coupled to the occurrence of spikes (Figures 7 and 8), rather than extended oscillations. Second, the time-frequency uncertainty principle limits the number of different frequency bands that can be used over a given period (for example, if computation takes place over 100 ms, the center frequencies must be more than 10 Hz apart). In addition, elaborate filtering mechanisms would be required for such coding schemes. Finally, because the LFP power follows a “1/f” spectrum (Figure 1C, F, and I), the raw power at high-gamma frequencies is extremely small, typically less than 1% of the total signal energy. So if the broadband response is due to multiple oscillators at different frequencies, their power will be too weak to support reliable communication channels.

### Relationship between Spikes and High-Gamma Power

Our results are consistent with several studies that have reported a correlation between spiking activity and LFP power at gamma and high-gamma frequencies [6]–[8],[17]–[19]. In addition, recent studies have revealed that low-frequency (<10 Hz) phase is also a significant predictor of the multiunit activity [7],[17],[19],[31],[32]. These studies used natural movies as the stimulus, which allowed them to study the fluctuations in the LFP at very low frequencies and over long time periods. In our study, the stimuli were presented in a periodic fashion (400 ms on, 600 ms off), and the average LFP locked to the stimulus onset was dependent on the stimulus size (Figure S8). In particular, after the stimulus was switched off at 400 ms, the evoked response showed a positive deflection whose amplitude depended on the size, followed by a sustained depression that was also dependent on size (Figure S8). Such slow changes likely reflect network dynamics not directly related to neuronal firing properties, because firing rates returned to spontaneous condition within 100 ms of stimulus offset. It is beyond the scope of this article to determine the neural mechanisms behind such characteristic changes in the evoked LFP, although such changes are likely to be reflected in the phase and amplitude of very low frequencies (at least up to a few Hertz) and carry information about the stimulus size.

### Relationship between Spikes and Gamma Rhythm

It is well known that both the magnitude and center frequency of the gamma rhythm depend critically on stimulus properties, such as contrast [23],[33], orientation [34]–[36], size [20], speed [37],[38], direction [6], and cross-orientation suppression [34]. Based on these results, there does not appear to be a straightforward relationship between the overall spiking activity of the network and the strength of the gamma rhythm. For example, firing rate decreases but gamma power increases with increasing stimulus size [20]. However, firing rates and gamma power co-vary with stimulus orientation [34]–[36]. Several mechanisms have been proposed for the generation of gamma rhythms, typically involving a network of inhibitory interneurons with or without reciprocal connections with pyramidal cells (ING and PING networks; [39]–[43], for reviews see [44],[45]). In addition, specialized types of layer 2/3 pyramidal neurons called “chattering cells” have also been reported to be involved in the generation of gamma oscillations [46]. These network mechanisms (that determine the magnitude of the gamma rhythm) may differ from the mechanisms that produce the majority of local multiunit activity.

We note, however, that the power in the gamma band between 30 and 80 Hz is not dependent only on the gamma “rhythm,” which may be weak or absent for a variety of stimulus conditions (e.g., low contrast, low spatial frequency, null orientation, small size). For example, Figure 1B, 1E, and 1H show an increase in power in a broad frequency range (>10 Hz), including the gamma range, in the first 100 ms after stimulus onset, before the onset of the gamma rhythm at ∼50 Hz. This broadband power might reflect synaptic activity [47]–[49], spike afterpotentials [50], or the spiking activity at higher frequencies (as shown in Figure 7, also discussed in the next section). Thus, it is crucial to dissociate between the band-limited gamma rhythm and the broadband increase in power (which includes the gamma band) due to synaptic and spiking events when assessing a functional role of gamma rhythms in cognitive processing.

### Nested Cross-Frequency Coupling

Several studies have shown that oscillations in different frequency bands of the LFP may be correlated with each other (for example, high-frequency power could be correlated with the phase at a lower frequency) and have hypothesized that this coupling could facilitate cortical processing simultaneously at several distinct timescales [3],[16],[51]–[53]. A recent study even shows complex phase-amplitude cross-frequency interactions in the absence of oscillatory peaks in the signal [5]. Our results are not inconsistent with these hypotheses, especially when one of the rhythms is at a lower frequency such as in the delta or theta range. However, at higher frequencies, such as gamma or high-gamma bands, it is important to distinguish between a “nested gamma rhythm” and possible contributions from phase-locked spikes.

An important issue here is the way the LFP signal is decomposed for time-frequency analysis. Most analysis techniques (Short Time Fourier Transform, multitaper analysis, etc.) necessarily decompose the LFP into a series of narrow band signals at various frequencies. In MP analysis, we start with an over-complete dictionary of functions that include both oscillatory (narrow-band) as well as transient (broadband) functions and find those that best represent the signal. We find that the LFP has several “broadband” components, such as the transients observed in the first ∼100 ms after stimulus onset or the sharp negativity associated with spikes, which are best described by either delta functions or a Gaussian with a small sigma. However, if such components are decomposed using traditional methods, we obtain a series of oscillatory functions whose amplitude and phase values are correlated (for example, the Fourier Transform of a delta function gives constant amplitude and zero phase at all frequencies). In other words, broadband components associated with spiking, stimulus onset, or eye movements [54], if decomposed into a series of oscillatory components, can lead to spurious correlations between those components.

### Population Dynamics at Fine Spatial Scales

Recent studies have argued that LFP has a spatial spread of ∼250 µm in cortex [55],[56]. Coupled with our results, this suggests that high-gamma activity is a sensitive measure of population firing rate of a small region near a microelectrode. Further, Figure 8 shows that changes in correlation in the neural population (in this case, the degree of gamma phase-locking) could also be reflected in the high-gamma range. The dependence of high-gamma power on the degree of synchronization/correlation in the neural population is expected to increase with the size of the neural population [8],[49]. Several cognitive mechanisms, such as selective attention, change the degree of correlation in the neural population [57],[58]; high-gamma activity potentially could be used to study these network dynamics at a fine spatial scale.

## Materials and Methods

Two separate datasets were used in this article. The first set was used to study the effect of size (the “size study,” all figures except Figure 5) on LFP power. The second set was used to study the effect of temporal frequency (the “temporal frequency study,” Figure 5). The behavioral task (described below) was the same for both datasets.

### Behavioral Task and Recording

The animal protocols used in this study were approved by the Institutional Animal Care and Use Committee of Harvard Medical School. Recordings were made from two male rhesus monkeys (*Macaca mulatta*, 11 and 14 kg). Before training, a scleral search coil and a head post were implanted under general anesthesia. After monkeys learned the behavioral task (∼4 mo), we implanted a 10×10 array of microelectrodes (Blackrock Microsystems, 96 active electrodes) in the right V1 (about 15 mm anterior to the occipital ridge and 15 mm lateral to the midline). The microelectrodes were 1 mm long and 400 µm apart, with impedance between 0.3 and 1 MΩ at 1 kHz. The entire length of the microelectrodes was inserted into cortex; we expect them to be in layer 2/3 or 4. Histology has not been performed. The receptive fields of the neurons recorded from the microelectrodes were in the lower left quadrant of the visual field at an eccentricity of about 3–5°.

Each monkey was trained to do an orientation-change detection task (Figure S1A). The monkey was required to hold its gaze within 1° of a small central dot (0.05–0.10° diameter) located at the center of a CRT video display (100 Hz refresh rate, 1,280×768 pixels, gamma corrected), while two achromatic odd-symmetric stimuli were synchronously flashed for 400 ms with an interstimulus period of 600 ms. For the size study, the stimulus in the left hemifield was a grating of variable size centered on the receptive field of one of the recording sites (new location for each session); the second stimulus was a Gabor stimulus with an SD of 0.5° located at an equal eccentricity on the opposite side of the fixation point. The monkey was cued to attend to the Gabor stimulus outside the receptive field, whose contrast was fixed at a low value to make the task demanding. Stimulus features (size and orientation) at the unattended location inside the receptive field were varied for each stimulus presentation in a pseudo-random order. At an unsignaled time drawn from an exponential distribution (mean 3,000 ms, range 0 to 7,000 ms for Monkey 1; 1,000 to 7,000 ms for Monkey 2), the orientation of the stimulus at the cued location changed by 90°. The monkey was rewarded with a drop of juice for making a saccade to the location of the changed stimulus within 500 ms of the orientation change. To account for saccade latency and to avoid rewarding the monkey for guessing, the monkey was rewarded only for saccades beginning at least 70 ms after the orientation change. Trials were truncated at 8,000 ms if the target had not appeared (∼5% of trials), in which case the animal was rewarded for maintaining fixation up to that time.

For the size study, the gratings were static with a spatial frequency of 4 cycles/degree (CPD), ∼100% contrast, located at the center of the receptive field of one of the sites (different recording site each session), at one of six different orientations (0°, 30°, 60°, 90°, 120°, and 150°) and six different radii (0.3°, 0.72°, 1.14°, 1.56°, 1.98°, and 2.4°), chosen pseudo-randomly. The Gabor stimulus outside the receptive field was also static, with a spatial frequency of 4 CPD, a fixed orientation (typically the preferred orientation of the recorded site) and size (SD: 0.5°), and an average contrast of ∼6% and ∼4.3% for Monkeys 1 and 2. The two monkeys performed the task in 10 and 24 recording sessions.

For the temporal frequency study (Figure 5), we used a counter-phasing Gabor stimulus inside the receptive field, with a spatial frequency of 4 CPD, preferred orientation, ∼100% contrast, SD of 0.8° and 1° for Monkeys 1 and 2, at five temporal frequencies—0, 0.62, 1.25, 2.5, and 5 Hz—for Monkey 1, and nine frequencies—0, 0.62, 1.25, 2.5, 5, 10, 20, 40, and 50 Hz—for Monkey 2. The Gabor stimulus outside the receptive field was static, with a spatial frequency of 4 CPD, preferred orientation, SD of 0.5°, and an average contrast of ∼3% and ∼7% for Monkeys 1 and 2. The two monkeys performed the task in 7 and 16 recording sessions.

Only correct trials were used for analysis. Catch trials (trials in which the orientation did not change) were excluded. For each correct trial, only the second stimulus through to the last stimulus before the target were used for analysis, so that the stimulus conditions were identical for the entire dataset. The first stimulus in each correct trial, which typically produced a stronger response, was analyzed separately, and similar results were obtained. For the size study, the average number of repetitions for each combination of size and orientation was 19 (range 6 to 36) for Monkey 1 and 15 (range 7 to 28) for Monkey 2. For the temporal frequency study (Figure 5), the average number of repetitions per temporal frequency was 82 (range 31 to 169) and 14 (range 6 to 40) for Monkeys 1 and 2.

Local field potential (LFP) and multiunits were extracted using commercial hardware and software (Blackrock System). Raw data were filtered between 0.3 Hz (Butterworth filter, 1^st^ order, analog) and 500 Hz (Butterworth filter, 4^th^ order, digital) to extract the LFP, and digitized at 2 kHz (16 bit resolution). Multiunits were extracted by filtering the raw signal between 250 Hz (Butterworth filter, 4^th^ order, digital) and 7,500 Hz (Butterworth filter, 3^rd^ order, analog) followed by an amplitude threshold.

### Receptive Field Mapping and Electrode Selection

Receptive fields were estimated by flashing small Gabor stimuli (SD of 0.05–0.1°) on a 9×9 (Monkey 1) or 11×11 (Monkey 2) rectangular grid that spanned the receptive fields of all the electrodes, while the monkeys attended to a Gabor stimulus outside the receptive field. The evoked LFP responses and the multiunit responses at different stimulus locations were fitted separately with a 2-D Gaussian to estimate the receptive field centers and sizes. Receptive fields obtained from multiunit and LFP responses were very similar. As the multiunit activity was more variable across days (and sometimes absent), we used the receptive field estimates from evoked LFP responses for analysis. For Monkey 1, the upper half of the grid did not yield any responses at all. Stable estimates of the receptive field centers (SD less than 0.1° across days) were obtained from 27 electrodes in Monkey 1 and 66 electrodes in Monkey 2. The remaining electrodes yielded weak and inconsistent evoked responses and were excluded from analysis.

For each recording session only the electrodes with receptive field centers within 0.2° of the stimulus center were used for analysis. For the size study, this yielded 56 electrodes (24 unique electrodes—many electrodes were recorded on multiple sessions) for Monkey 1 and 138 electrodes (66 unique) for Monkey 2. Out of these, we selected electrodes for which the average firing rate between 200 and 400 ms (the period over which analysis was done, see below) was at least 1 spike/s for all stimulus sizes, and the signal-to-noise ratio of the isolation was greater than 1.5. This yielded 15 (11 unique) and 104 (58 unique) “spike” electrodes for Monkeys 1 and 2, respectively. For the temporal frequency study, 44 (22 unique) and 90 (59 unique) electrodes had receptive fields within 0.2 degrees of the stimulus center. Out of these, we selected electrodes for which the average firing rate between 200 and 400 ms was at least 1 spike/s at zero temporal frequency, and the signal-to-noise ratio of the isolation was greater than 1.5. This yielded 19 (13 unique) and 66 (42 unique) spike electrodes for Monkeys 1 and 2, respectively.

To account for the multiplicity of some electrodes in our dataset, all analyses were repeated after pooling the data from the same electrode across days. Similar results were obtained.

### Data Analysis

#### Time-frequency analysis

Time-frequency decomposition was performed using the MP algorithm [59]. MP is an iterative procedure to decompose a signal as a linear combination of members of a specified family of functions g_γn_, which are usually chosen to be sine-modulated Gaussians—that is, Gabor functions or “Gabor atoms”—because they give the best compromise between frequency and time resolution. In this algorithm, a large overcomplete dictionary of Gabor atoms is first created. In the first iteration the atom g_γ0_ that best describes the signal *f(t)* (i.e., has the largest inner product with it) is chosen from the dictionary and its projection onto the signal is subtracted from it. The procedure is repeated iteratively with the residual replacing the signal. Thus, during each of the subsequent iterations, the waveform g_γn_ is matched to the signal residue R^n^f, which is the residue left after subtracting the results of previous iterations. Mathematical details of this method are presented elsewhere [18]. Time-frequency plots were obtained by calculating the Wigner distribution of individual atoms and taking the weighted sum [59].

In MP, by choosing a large dictionary of Gabor atoms, we get fewer a priori limitations on decomposition and more free parameters than other methods and are able to detect local patterns in the signal with the best possible compromise between time and frequency resolution. Because the overcomplete dictionary has basis functions with a wide range of time and frequency support, we can represent rhythms (alpha, gamma, etc.) with functions that are extended in time but narrow in frequency, as well as transients (due to spiking or stimulus onset) with functions that are brief in time but broad in frequency. The availability of “broadband” basis functions that can capture the sharp transients associated with spikes (Figures 7 and 8) is critical for the results shown in this article. Further details about this method and its advantages over traditional methods such as Short Time Fourier Transform are discussed elsewhere [8],[18]. In particular, we discuss why this method is much better suited to study the high-gamma activity in Supplementary Discussion 3 of [8]. We have made the software used for MP computation available online at http://erl.neuro.jhmi.edu/mpsoft.

MP was performed on signals of length 4,096 (−1,148 ms to 900 ms at 0.5 ms resolution, where zero denotes the time of stimulus onset), yielding a 4,096×4,096 array of time-frequency energy values (with a time resolution of 0.5 ms and frequency resolution of 2,000/4,096 Hz = ∼0.5 Hz).

Power versus frequency plots (Figure 1C, 1F, 1I) were generated by averaging the energy within a time period at a given frequency.
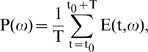
(1)where E(t,ω) is the mean energy averaged over trials at time t and frequency ω obtained from the MP algorithm. When showing population data (Figure 1F, 1I), we averaged the log_10_(P(ω)) values of individual sites. The power was shown either between 200 and 400 ms (t_0_ = 200, T = 200) or during baseline (t_0_ = −300, T = 300).

Time-frequency difference plots (Figures 1B, 1E, 1H, 4A, 4C, 5A, 5C) were obtained using the following equation:
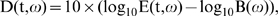
(2)where B(ω) is the baseline energy as defined in equation 1 with t_0_ = −300 ms, T = 300 ms. For the population data, we averaged the D(t,ω) values of individual sites.

Multitapering analysis [60] was performed with three tapers, implemented in Chronux 2.0 [61], an open-source, data analysis toolbox available at http://chronux.org. Spectrum and spectrogram were computed using functions mtspectrumc and mtspecgramc in Chronux, respectively. Essentially, the multitaper method reduces the variance of spectral estimates by pre-multiplying the data with several orthogonal tapers known as Slepian functions. Details and properties of this method can be found here [21],[22].

#### Cross-correlation analysis (Figures 4 and 5)

We adopted the method used by Womelsdorf and colleagues [12] based on Spearman-rank correlation to compute the cross-correlation between firing rates and power in different frequency bands. As a measure of the cross-correlation at time lag L, we computed the Spearman-rank correlation between the power between −100 and 500 ms and the firing rates from L−100 to L+500 ms (both quantities were computed with a time resolution of 2 ms; thus, we obtained 300 data pairs). This method is approximate because the power and rate values are not independent across time. However, the Spearman rank correlation analysis avoids assumptions about the underlying distributions [12]. Note that correlation does not change if values are scaled by a constant. For example, scaling down the green traces shown in Figure 4B and 4D (for the gamma band) would appear to improve their alignment with the black trace, however it will not change the Spearman correlation. We obtained cross-correlation functions for time lags (L) between −20 and 20 ms. Because the results did not vary greatly as a function of L and were maximum near L = 0, we report only the values for L = 0 in the main text.

#### Behavior and eye positions

The behavioral task was demanding and required sustained attention on the stimulus. Monkey 1 was correct in 78% of the completed trials (5% missed, 17% false alarms) for the size study and 78% (6% missed, 16% false alarms) for the temporal frequency study. Monkey 2 was correct in 93% of the completed trials (4% missed, 3% false alarms) for the size study and 90% (6% missed, 4% false alarms) for the temporal frequency study. Average eye positions, monitored at 200 Hz using a scleral search coil, differed by less than 0.03° across conditions, for both the size and temporal frequency studies, for both monkeys.

## Supporting Information

Figure S1 Task and stimuli. (A) Task design. Monkeys were trained to an orientation-change detection task. The monkey was required to hold its gaze within 1° of a small central dot (white central dot), while two achromatic odd-symmetric stimuli were synchronously flashed for 400 ms with an interstimulus period of 600 ms. One was a grating of different sizes and orientations, centered on the receptive field of one of the recording sites (red circle; receptive fields of all the electrodes were in the lower left quadrant at an eccentricity of 3–5°); the second stimulus was a Gabor with a fixed size and orientation located at an equal eccentricity in the other hemifield. The monkey was cued to attend to the Gabor stimulus outside the receptive field. At an unsignaled time drawn from an exponential distribution, the orientation of this stimulus changed by 90°. The monkey was rewarded with a drop of juice for making a saccade to this stimulus within 500 ms of the orientation change. (B) The three gratings whose time-frequency plots are shown in Figure 1B, along with the mean receptive field size of the sites (red ellipse).
(0.72 MB TIF)

Figure S2 Same analysis as Figure 2, when the spectra in (A) are computed using the multitaper method (with three tapers). The signal is taken between 200 and 400 ms with no zero padding, which yields a frequency resolution of 5 Hz.
(0.24 MB TIF)

Figure S3 Same analysis as shown in Figure 4, when the time-frequency power spectra were computed using the multitaper method. The windows were 128 ms (A and C) or 64 ms (B and D) ms long and were shifted by 2 ms.
(2.21 MB TIF)

Figure S4 Correlations between firing rate and LFP power in different frequency bands for stimuli presented at high temporal frequencies. (A) Average time-frequency energy difference plots (left panel) and changes in LFP power as well as firing rates from baseline (right panels), for a stimulus frequency of 10 Hz (contrast profile shown in red on top of the right panels), for 66 sites in Monkey 2. Same format as in Figure 5. (B) Same as (A) but for a temporal frequency of 20 Hz.
(1.63 MB TIF)

Figure S5 Same analysis as in Figure 6, done using the multitaper method with three tapers.
(0.64 MB TIF)

Figure S6 Spike-field coherence (SFC), computed between 150 and 406 ms after stimulus onset, for the six stimulus sizes. (A) Average SFC when spikes and LFP were taken from the same electrode, for 15 pairs in Monkey 1. (B) Average SFC of 85 spike-LFP pairs in Monkey 1, taken from separate electrodes. Both electrodes were within 0.2° of the stimulus center. (C–D) Same as (A–B), but for 104 and 563 spike-LFP pairs for Monkey 2.
(0.25 MB TIF)

Figure S7 STTFA analysis using the multitaper method. (A) The left plot shows the nSTTFA for Monkey 1 during baseline period (similar to the right column in Figure 7B), when the time-frequency power spectrum is computed using multitaper method (window length = 64 ms, window shift = 0.5 ms). The right plot shows the nSTTFA computed from spikes between 200 and 400 ms when the largest stimulus was presented (similar to the right column in Figure 8B). (B) Same analysis as (A), with a window of 32 ms. (C,D) Same as (A,B), for Monkey 2.
(1.66 MB TIF)

Figure S8 Evoked LFP response, computed by averaging the LFP traces locked to the stimulus onset. The black horizontal line represents the stimulus period. The low magnitude high-frequency oscillations observed in some of the traces are due to the refresh rate of the monitor at 100 Hz.
(0.25 MB TIF)

## Abbreviations

LFP: local field potential
MP: Matching Pursuit
rSTTFA: randomized spike-triggered time-frequency average
SFC: spike-field coherence
STA: spike-triggered average
STTFA: spike-triggered time-frequency average
