# Supplementary figures and images for: Different Origins of Gamma Rhythm and High-Gamma Activity in Macaque Visual Cortex
Source: PLoS Biol. 2011 Apr 12;9(4):e1000610. doi: 10.1371/journal.pbio.1000610 (PMC3075230; doi:10.1371/journal.pbio.1000610)

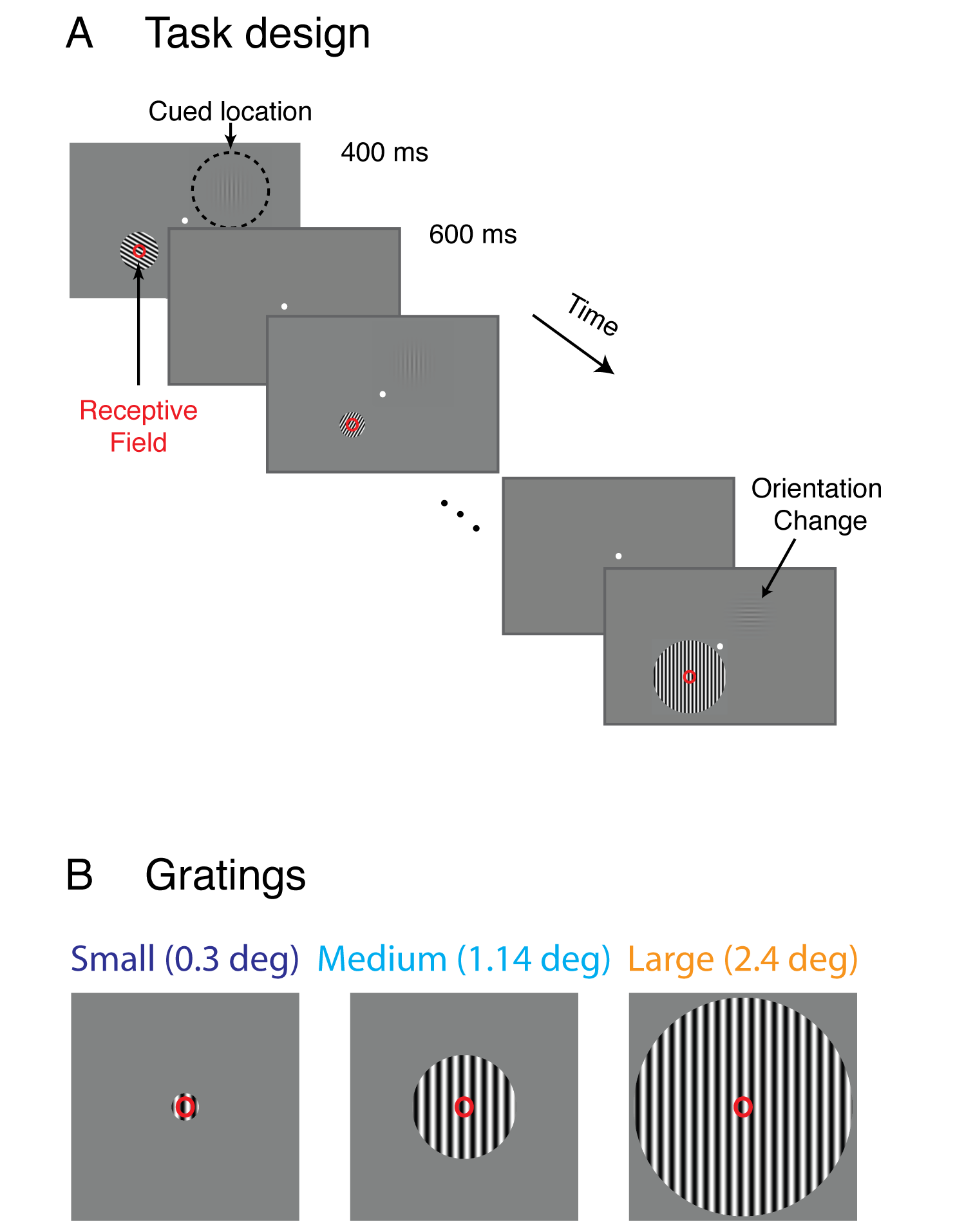

Supplement: Figure S1 — Task and stimuli. (A) Task design. Monkeys were trained to an orientation-change detection task. The monkey was required to hold its gaze within 1° of a small central dot (white central dot), while two achromatic odd-symmetric stimuli were synchronously flashed for 400 ms with an interstimulus period of 600 ms. One was a grating of different sizes and orientations, centered on the receptive field of one of the recording sites (red circle; receptive fields of all the electrodes were in the lower left quadrant at an eccentricity of 3–5°); the second stimulus was a Gabor with a fixed size and orientation located at an equal eccentricity in the other hemifield. The monkey was cued to attend to the Gabor stimulus outside the receptive field. At an unsignaled time drawn from an exponential distribution, the orientation of this stimulus changed by 90°. The monkey was rewarded with a drop of juice for making a saccade to this stimulus within 500 ms of the orientation change. (B) The three gratings whose time-frequency plots are shown in Figure 1B, along with the mean receptive field size of the sites (red ellipse). (0.72 MB TIF) [file pbio.1000610.s001.tif]

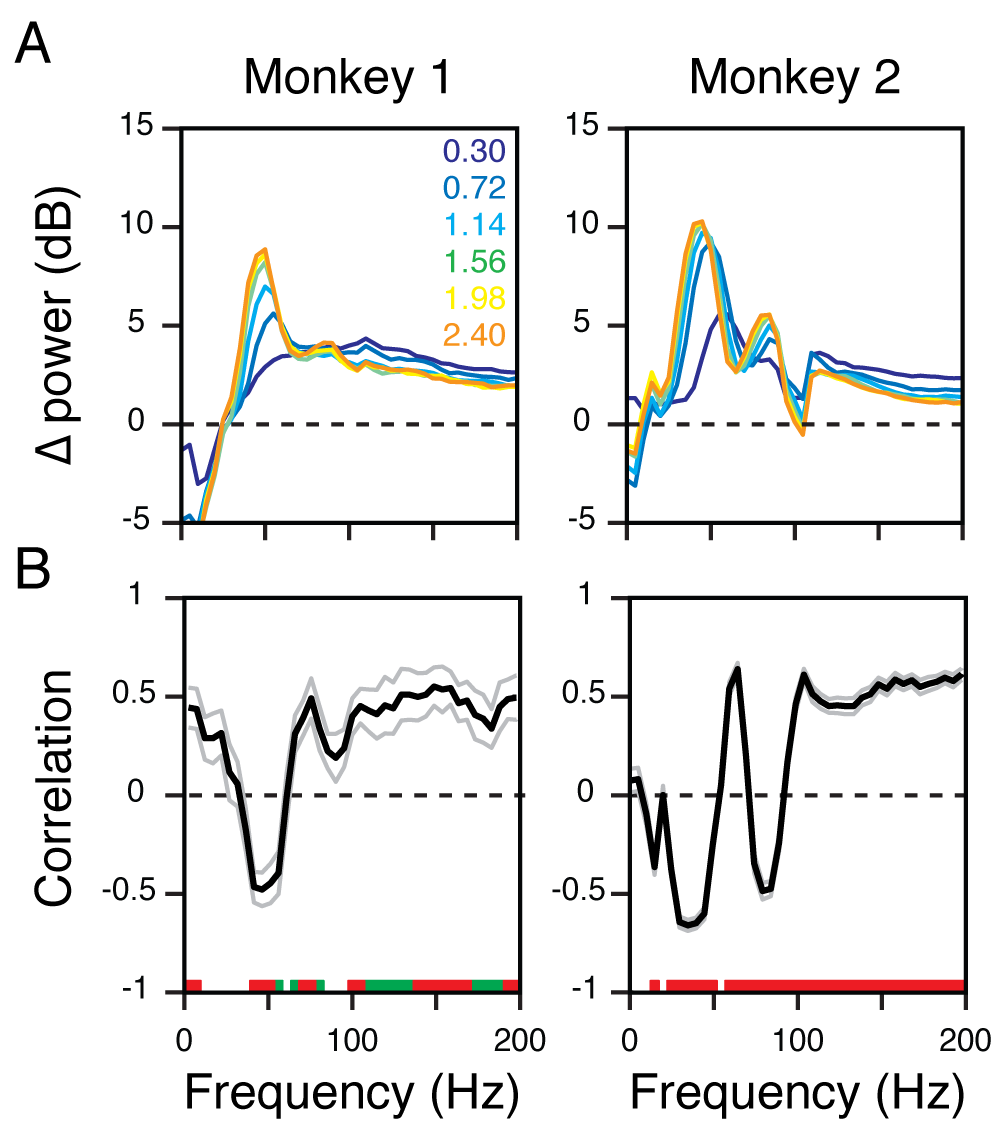

Supplement: Figure S2 — Same analysis as Figure 2, when the spectra in (A) are computed using the multitaper method (with three tapers). The signal is taken between 200 and 400 ms with no zero padding, which yields a frequency resolution of 5 Hz. (0.24 MB TIF) [file pbio.1000610.s002.tif]

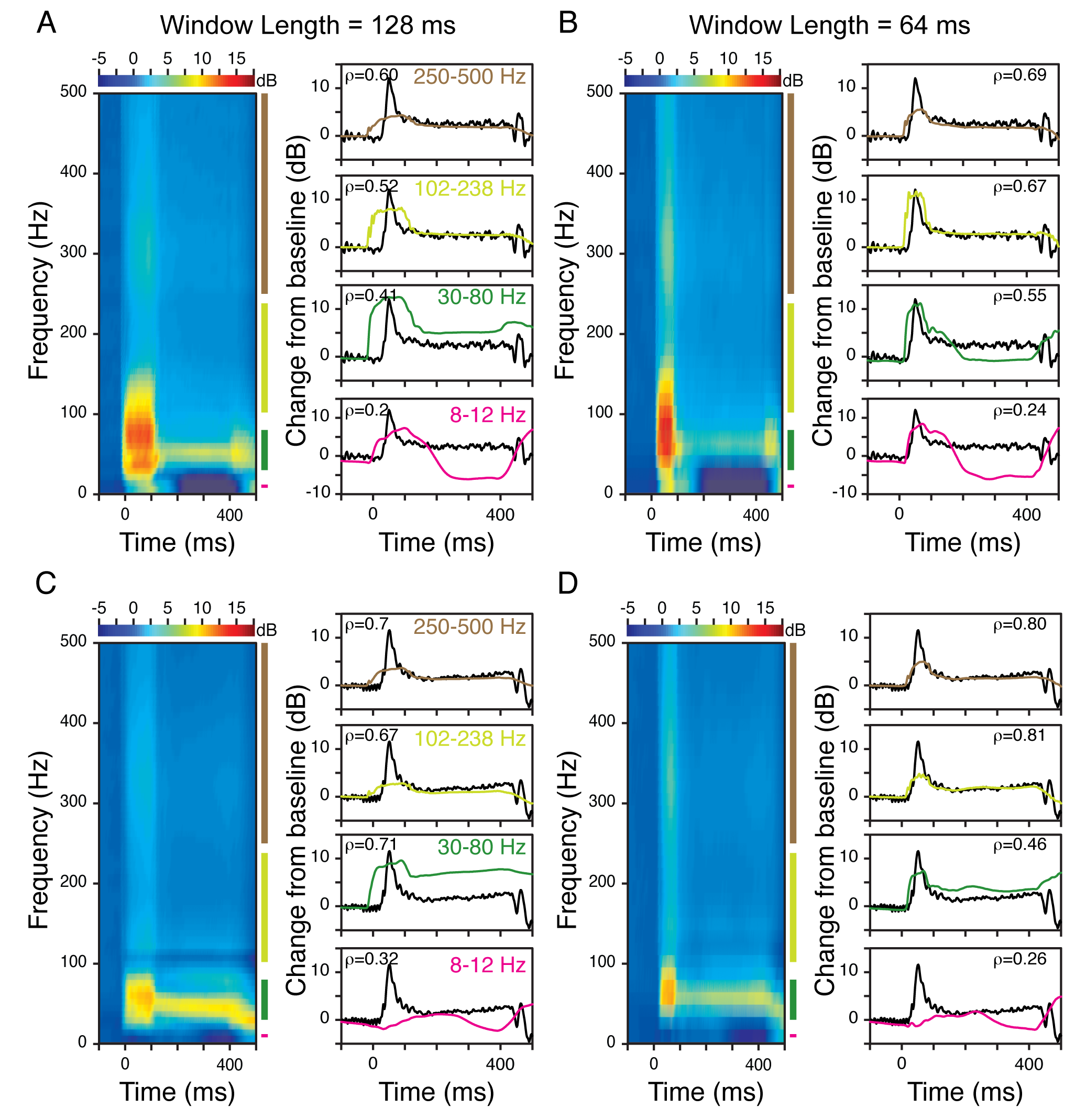

Supplement: Figure S3 — Same analysis as shown in Figure 4, when the time-frequency power spectra were computed using the multitaper method. The windows were 128 ms (A and C) or 64 ms (B and D) ms long and were shifted by 2 ms. (2.21 MB TIF) [file pbio.1000610.s003.tif]

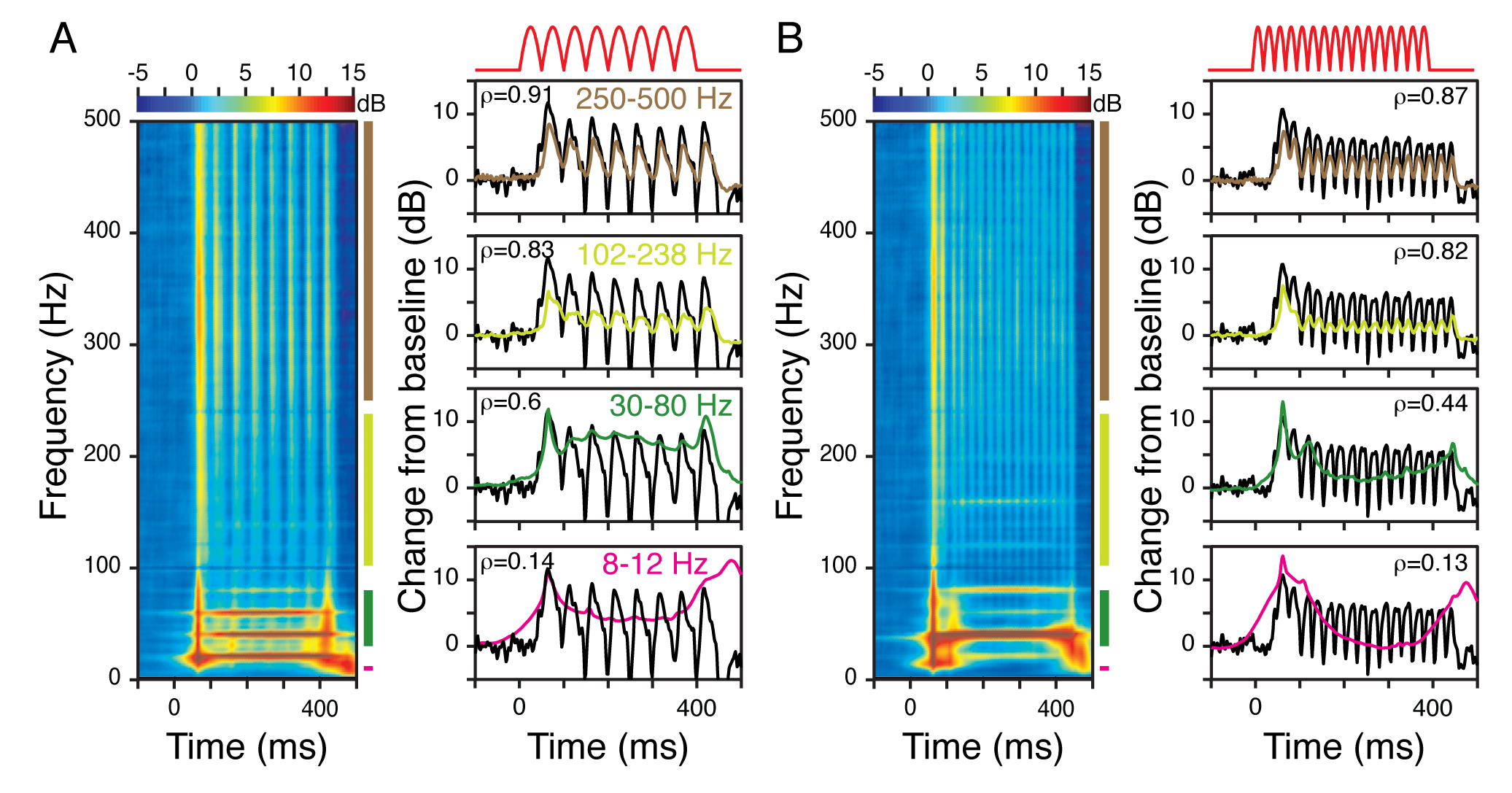

Supplement: Figure S4 — Correlations between firing rate and LFP power in different frequency bands for stimuli presented at high temporal frequencies. (A) Average time-frequency energy difference plots (left panel) and changes in LFP power as well as firing rates from baseline (right panels), for a stimulus frequency of 10 Hz (contrast profile shown in red on top of the right panels), for 66 sites in Monkey 2. Same format as in Figure 5. (B) Same as (A) but for a temporal frequency of 20 Hz. (1.63 MB TIF) [file pbio.1000610.s004.tif]

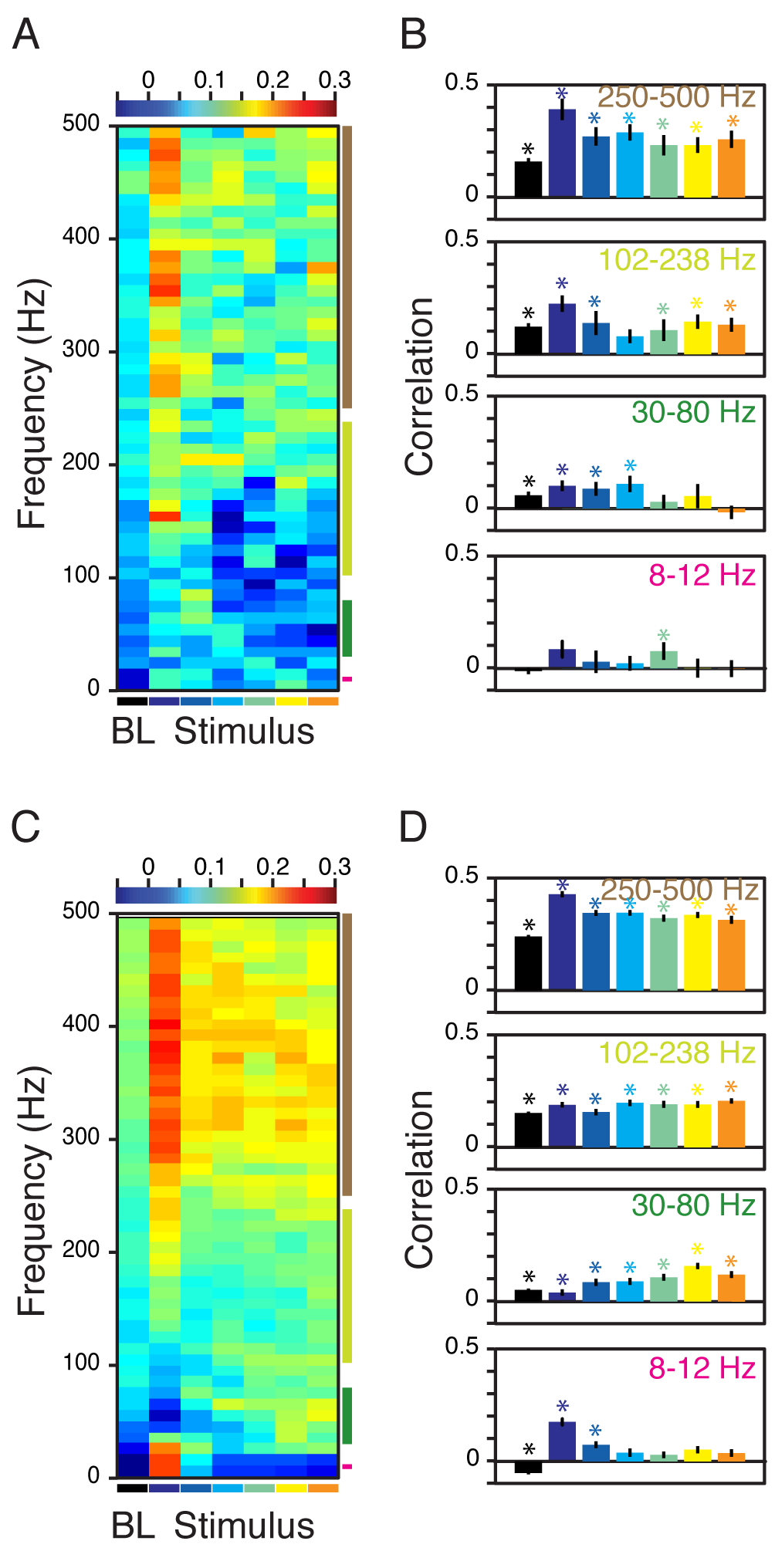

Supplement: Figure S5 — Same analysis as in Figure 6, done using the multitaper method with three tapers. (0.64 MB TIF) [file pbio.1000610.s005.tif]

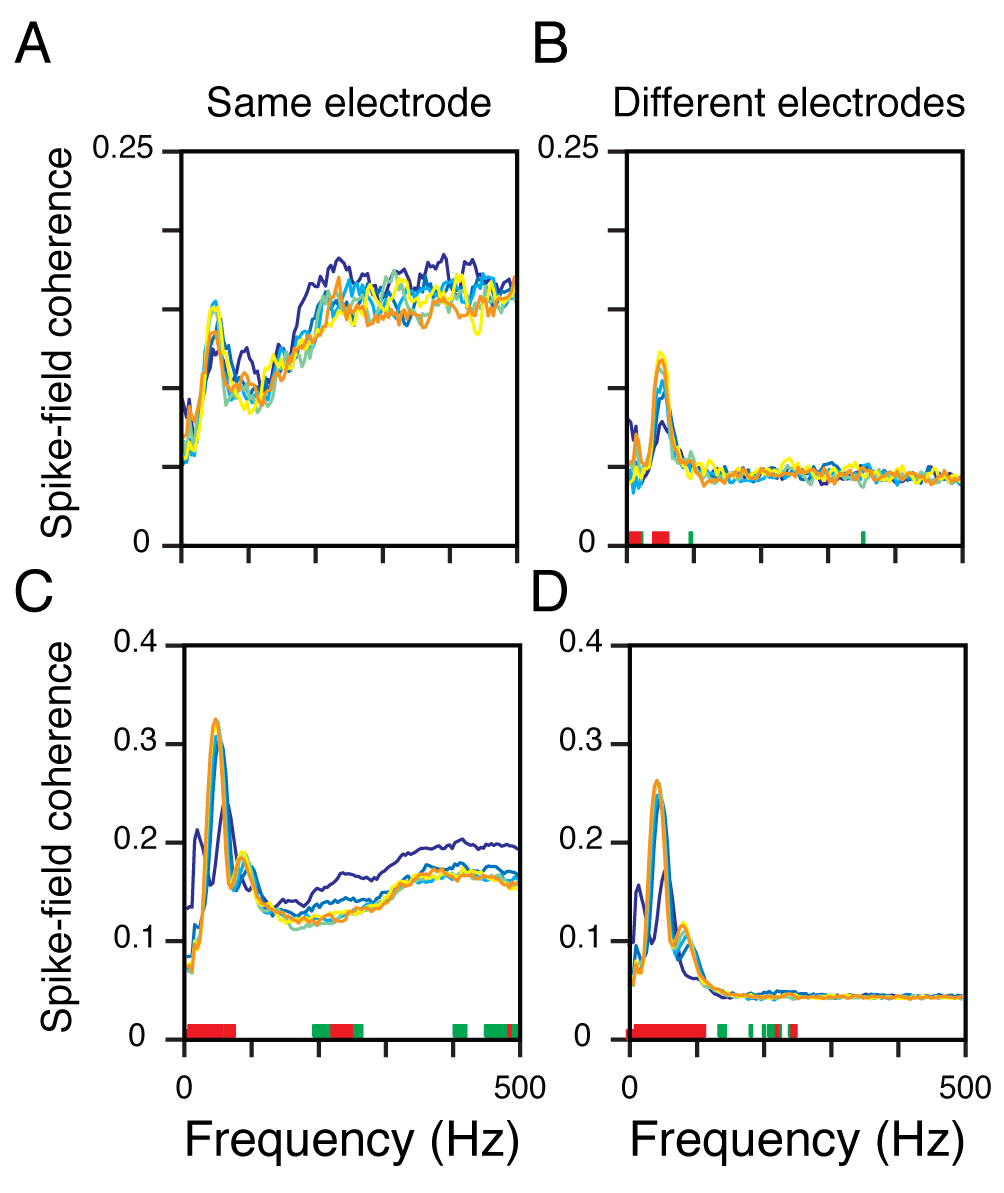

Supplement: Figure S6 — Spike-field coherence (SFC), computed between 150 and 406 ms after stimulus onset, for the six stimulus sizes. (A) Average SFC when spikes and LFP were taken from the same electrode, for 15 pairs in Monkey 1. (B) Average SFC of 85 spike-LFP pairs in Monkey 1, taken from separate electrodes. Both electrodes were within 0.2° of the stimulus center. (C–D) Same as (A–B), but for 104 and 563 spike-LFP pairs for Monkey 2. (0.25 MB TIF) [file pbio.1000610.s006.tif]

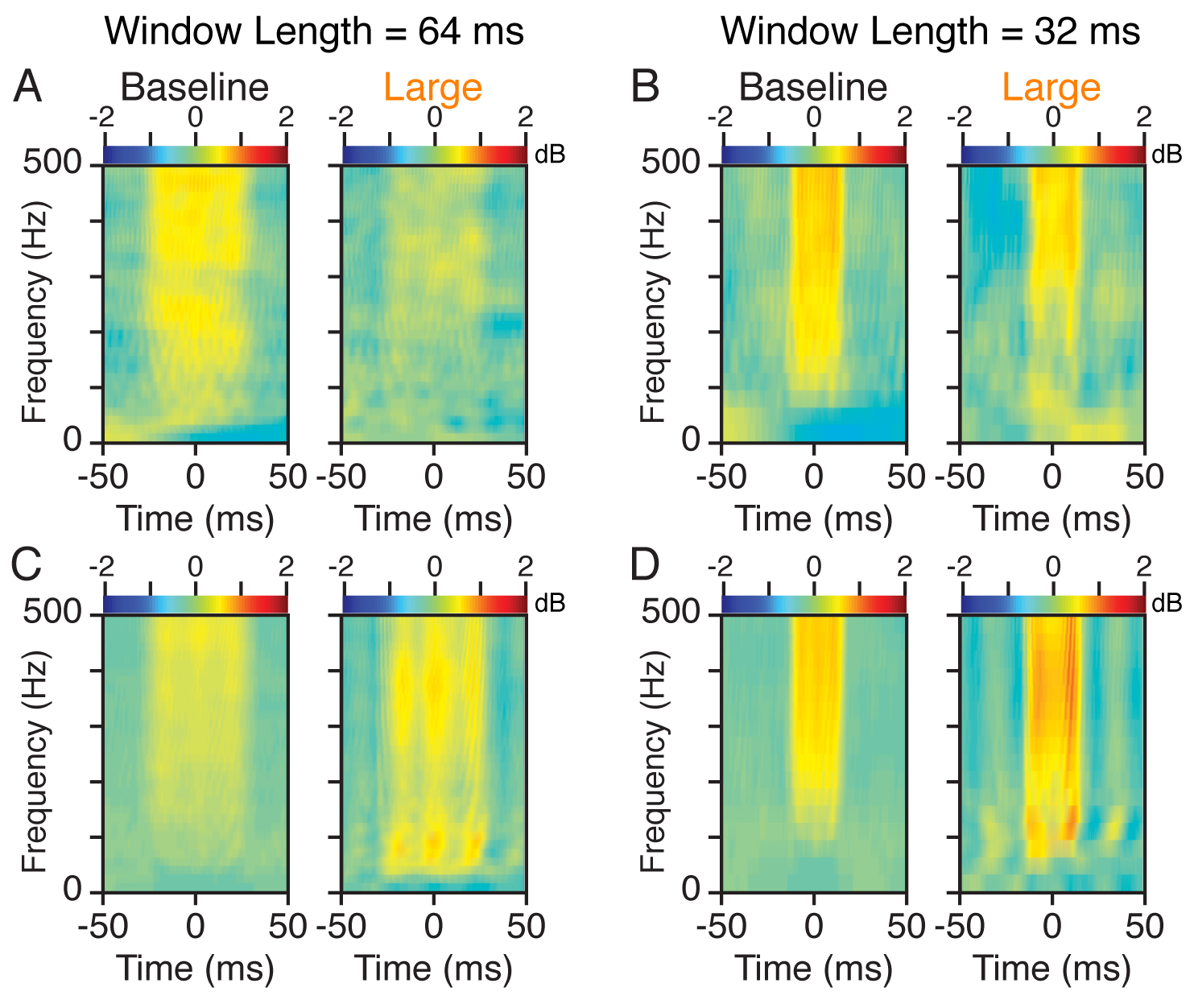

Supplement: Figure S7 — STTFA analysis using the multitaper method. (A) The left plot shows the nSTTFA for Monkey 1 during baseline period (similar to the right column in Figure 7B), when the time-frequency power spectrum is computed using multitaper method (window length = 64 ms, window shift = 0.5 ms). The right plot shows the nSTTFA computed from spikes between 200 and 400 ms when the largest stimulus was presented (similar to the right column in Figure 8B). (B) Same analysis as (A), with a window of 32 ms. (C,D) Same as (A,B), for Monkey 2. (1.66 MB TIF) [file pbio.1000610.s007.tif]

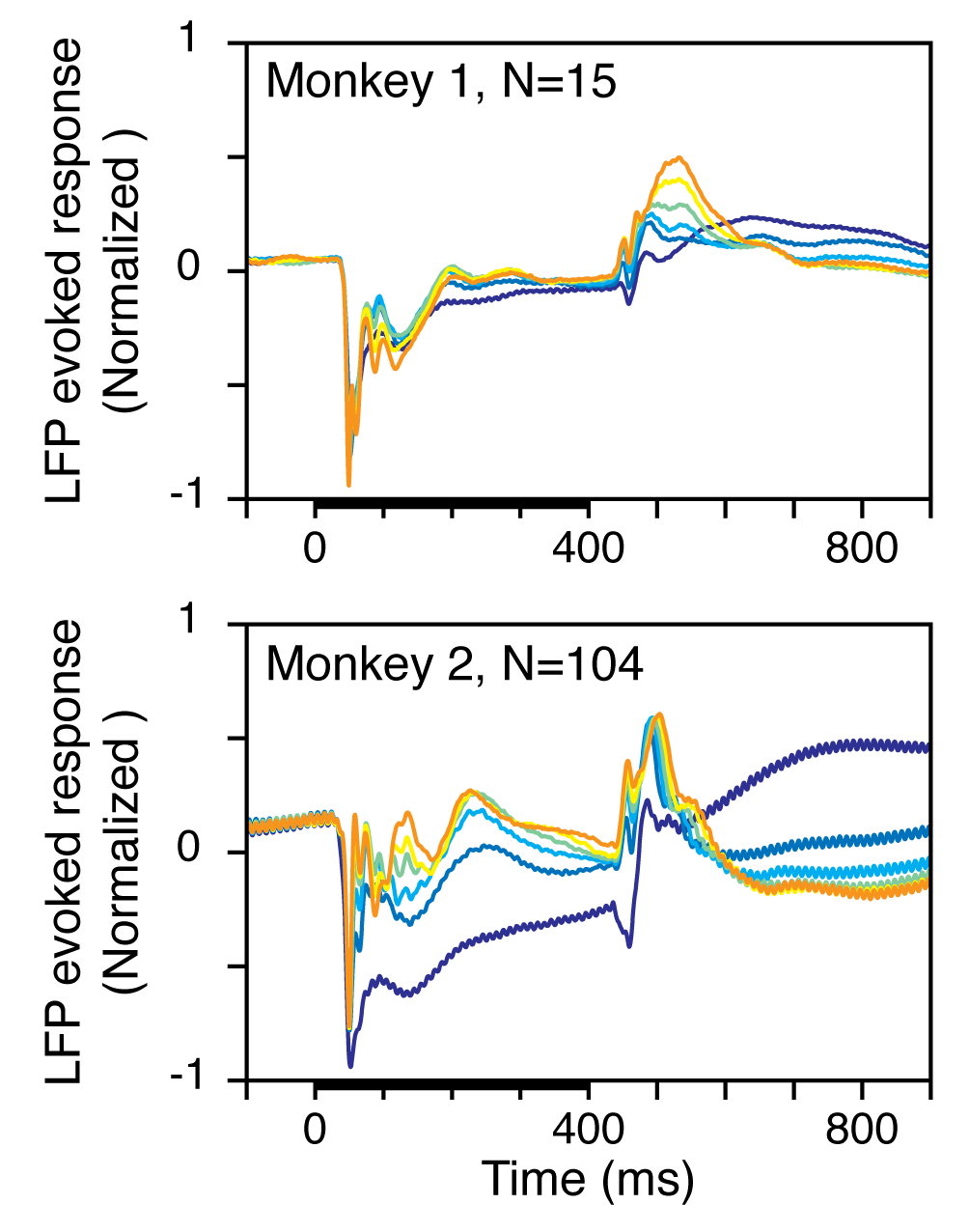

Supplement: Figure S8 — Evoked LFP response, computed by averaging the LFP traces locked to the stimulus onset. The black horizontal line represents the stimulus period. The low magnitude high-frequency oscillations observed in some of the traces are due to the refresh rate of the monitor at 100 Hz. (0.25 MB TIF) [file pbio.1000610.s008.tif]
